# Report of the Distribution Committee

**Published:** 1916-12-23

**Authors:** 


**KING EDWARD'S HOSPITAL FUND**—(*continued*).

### Report of the Distribution Committee.

(1) The Governors and General Council have this year fixed the sum available for distribution amongst the London hospitals at £162,500, as against £133,500 in 1915.

(2) This is the largest sum that has ever been distributed by the Fund amongst the hospitals, being an increase of £29,000 over the last two years and £11,500 over the previous maximum of £151,000 which was reached in the years immediately preceding the war.

(3) While the increase in total distribution over the previous highest amount is thus £11,500, the increase in grants in aid of maintenance is much greater, being £28,075. This is due to the postponement of building schemes and the consequent reduction of grants towards capital expenditure. Of the capital grants of £19,075 recommended this year only £6,525 is on account of schemes which have been begun since the outbreak of war on the ground of exceptional urgency: the balance has been devoted to relieving hospitals of capital liabilities incurred before the war.

### CONSIDERATIONS DETERMINING AWARDS.

(4) In distributing the increased grants to maintenance, the Committee have again been faced with the difficulty that it is impossible to make reliable comparisons between the financial positions of different hospitals except on the evidence of the audited accounts, which are necessarily those of 1915, when the effects of the war had not been felt as fully as they have been during 1916. They recognise, therefore, that the awards cannot actually represent the relative claims of the hospitals as they are at the present moment. They have, however, done whatever they could to distribute the increased grants in proportion to the probable urgency of need in 1916. Amongst other things, they have given rather greater weight than usual to the more permanent elements which affect a hospital's power to stand increased financial strain, such as the relative amount of fixed income and the relative amount of available reserve funds. But the actual results of 1916 cannot be taken into account until the next distribution.

(5) While thus giving special assistance to hospitals which, through no fault of their own, are financially weak, the Committee have maintained the position that the help of the Fund is not to be looked upon as a substitute for the efforts of the hospitals themselves, whether in raising funds or in prudent and economical management. Exceptional grants in reduction of debt, in particular, are not given without a careful consideration of the steps already taken by the hospitals concerned, and especially of the possibility of increased local support. In some neighbourhoods it is obvious that for the bulk of the necessary funds the hospital must depend upon external help. But even in these cases it is impossible for the Fund to do more than supplement the contributions raised by the hospitals themselves; while in other parts of London exceptional assistance must always be conditional upon sustained local as well as general efforts.

(6) The Committee have again taken into consideration the returns made by the hospitals of the work done in the treament of naval and military patients during the previous year, and also during the first nine months of the current year.

(7) The number of hospitals applying for grants is 107, being three in excess of last year.

(8) The position with regard to the amalgamation of the Throat, Nose, and Ear Hospitals, so far as the Fund is concerned, is the same as last year, except that an extra grant is recommended to the amalgamated Hospital for Diseases of the Throat to assist in meeting additional expenditure incurred in consequence of the postponement of the building scheme owing to the war.

### HOSPITALS' CO-OPERATION WITH PUBLIC AUTHORITIES.

(9) The Committee receive year by year evidence of the increasing work done by the voluntary hospitals (quite apart from the treatment of naval and military patients) in the cure or prevention of disease in co-operation with State or Municipal authorities. This is one of the numerous ways in which the hospitals take their part in the development of fresh methods of promoting public health. The attitude of the Fund towards these activities, as they affect its distribution, depends, generally speaking, upon their results as shown in the statistics of work done and in the relation between income and expenditure.

### KING EDWARD'S HOSPITAL FUND—(*continued*).

The latest development in this direction is the preparation by various hospitals of schemes for the treatment of venereal diseases in co-operation with the Local Government Board and the London County Council in accordance with the recommendations of the recent Royal Commission. These schemes naturally do not affect the accounts and statistics for 1915 or even, as a rule, 1916. But in one case, that of the London Lock Hospital, an application for a capital grant in aid of sanitary and other improvements at the Female Hospital has been received, and in view of the importance of increasing the efficiency of this hospital in these respects the Committee are recommending a substantial grant towards the sanitary improvements. The Committee further recommend that the Council, which has already by large grants enabled the Male Lock Hospital to be brought thoroughly up to date, should also offer substantial assistance towards an approved scheme for the reconstruction of the Female Lock Hospital.

APPEALS IN AID OF AFTER-WAR WORK.

(10) Both last year and the year before the Committee drew attention in their report to the fact that hospitals generally had, with the approval of the Fund, adopted the policy of deferring till after the war all schemes involving capital outlay unless of exceptional urgency. Several of the schemes thus postponed had already been submitted to the Fund in accordance with the well-known request of the Council to that effect. Other schemes have been prepared and submitted to the Fund as a preparatory step, although no action is contemplated until after the war.

Appeals have, however, been issued by some hospitals in aid of schemes for work to be carried out after the war which had not been submitted to the Fund; and, with the object of avoiding any misunderstanding and in fairness to hospitals that had, with the approval of the Fund, held over their appeals, the Committee issued a circular drawing attention to the various statements that had already been made by the Fund on the subject.

(11) The following grants made in 1913 in aid of schemes of building expenditure which have been deferred during the war again require to be recommended for renewal:

Queen Charlotte's Lying-in Hospital, £1,000 to new out-patient department.

St. Mary's Hospital for Women and Children, Plaistow, £1,500 to new nurses' home and out-patient department.

(12) While recognising that the cost of working at hospitals generally has been raised by the war, the Committee have specially considered all cases where the increase has been markedly greater than at similar hospitals. Where no adequate explanation is apparent they have appended a remark to their award.

(13) The Committee have continued the practice of recommending that extracts from the visitors' reports should, where desirable, be communicated privately to the hospitals with the grants. By this means the reports, in addition to their great value to the Committee in making the awards, are rendered of direct service to the hospitals concerned. The Committee desire to tender their best thanks to the visitors for their services in this connection.

The details of the awards to the various hospitals are appended.

For the Committee, W. S. CHURCH, Chairman.

December 1916.

